# Reports on Hospitals of the United Kingdom

**Published:** 1914-09-19

**Authors:** Henry Burdett


### REPORTS ON

### Hospitals of the United Kingdom.

By SIR HENRY BURDETT, K.C.B., K.C.V.O.

SERIES III.

### THE CUMBERLAND INFIRMARY, CARLISLE.

We inspected this institution in the beginning of 1893, and wrote a full account of it in THE HOSPITAL of February 25, 1893, page 351. At that time there was accommodation for 100 beds, the average number occupied being 78. The in-patients amounted to 750, and the out-patients to 1,778 each year. The income was £4,288, and the expenditure about £3,750. At that time the buildings consisted of an old central wing, erected about 1842, to which a pavilion of two storeys, containing four excellent wards, had been added. This pavilion of two storeys was divided in the centre by a central staircase of handsome proportions, from which a ward opened out on each side. These well-proportioned wards contained an abundance of air, light, cubic and superficial space. We found them in 1893 in a state of the most perfect order, with all the relative modern appliances at that time used, but with no slop sinks. The central wing, where the officers' quarters, kitchen, board-room, and other offices were situated, also contained a number of wards. The number of out-patients already given indicates that the out-patient wing in 1893 was small and without special features. The kitchen was situated in the basement, which also contained some very unsatisfactory sleeping accommodation for the servants, a practice common enough twenty years ago. We called attention to these matters, and strongly recommended that other and better accommodation should be provided. One marked feature was the homelike, comfortable atmosphere which prevailed throughout the whole institution. The resident staff were devoted and zealous, and the whole condition of the Cumberland Infirmary was marked by efficiency of administration.

### A SECOND INSPECTION.

On June 20, 1914, we again inspected this institution. We entered it with very pleasant anticipations, remembering what a bright, cheerful, and happy community it had been twenty-one years before, and we looked forward to finding it an improved and modern development, on, if possible, better lines, of what was one of the most attractive of the smaller county hospitals in 1893.

In the twenty-one years which had elapsed a new West Pavilion for twenty-four children (cots) and twelve male patients had been erected. New ward kitchens and bathrooms had been provided, with sanitary annexes, and a new clinical room to each ward. The old basement kitchen had been done away with, and a new kitchen, pantries, and other necessary provision to complete this unit had been added. A new ophthalmoscope-room, an $x$-ray department, a new nurses' home and conservatory, with additional nurses' rooms on the second floor, and new sanitary towers, had also been built. The heating and other arrangements had been improved by the erection of a boiler house, and the laundry had been reconstructed and made adequate to the needs of a larger hospital. The buildings as they stand to-day contain accommodation for 150 in-patients, but only 102 beds have been authorised for occupation, the average daily number of in-patients being 96. In 1913 the in-patients were 1,357 and the out-patients 3,902. It will thus be seen that the buildings as they at present stand are ample, if not excessive. The floors of the new wards are constructed of teak in squares, but the appearance is rather disappointing. They each contain central fireplaces, which appeared to us to be imperfect, for they led to too great a consumption of coal, gave relatively a small amount of heat, and the fires lacked brightness, having no circular frontage to the grates, which when present give a bright and cheerful appearance to the wards.

On the whole the wards were satisfactory, but we noticed that the pillows were, with the exception of a few flock, all of feathers, and that there were too few of them, as most of the beds seemed to have not more than one. It is desirable that the flock pillows should be done away with altogether, and that hair pillows, in addition to the feather pillows, should be supplied without delay. The nurses are at present housed comfortably. There is a light and attractive day-room, and the sleeping accommodation and its arrangements were good. We would suggest that a few good books of an interesting and readable type should be provided for the nurses' use. The sleeping accommodation for the domestic staff has been greatly improved; but, having regard to the number of this staff, and to the fact that the laundry-maids reside in the hospital, it is but reasonable and proper that a good, well-equipped recreation or sitting room should be set apart for their use. It seemed to us that a large and airy apartment, well suited (and probably originally provided) for the purpose, but now occupied as a needle-room, should be fitted up for the use of the domestic staff. The needle-room might perfectly well be provided elsewhere and without difficulty. In 1893 we found the mortuary to be a surprisingly bad one. The present mortuary has been erected on a defective plan, and has no view-room, which ought to be forthcoming quickly.

One of the consulting surgeons, Mr. Herbert W. Page, who has taken the deepest interest in this institution, and has had much to do with its enlargement, was kind enough to meet us at the infirmary, and we had the pleasure of being taken round by Miss Sylvia Parker, the matron.

### Something Wrong Somewhere.

Speaking of the hospital buildings as a whole, we should say that structurally the institution has been transformed for the better. Administratively, however, it seemed to us that it has been changed sadly for the worse. There is a great deal more in the atmosphere of a hospital than the outside world wots of. In the old days Cumberland Infirmary, as we have stated, was an inspiriting and helpful institution to visit, owing to the marked efficiency and individual interest and smartness which characterised it. We gathered the impression that at present there is little or no spirit or life in the place. There was a lack of smart, contented briskness in the general atmosphere and work going on in the wards. Cumberland Infirmary is one of those institutions which require some one in authority with technical knowledge and experience, who is constantly devoting attention and time to the supervision and enforcement of smartness throughout the wards and those portions of the establishment which are included in the hospital section. Good housekeeping is, of course, essential to promote the general comfort of the staff and patients, but the real happiness and efficiency of a hospital must depend largely upon the presence or absence of devotion and zeal, coupled with a keen interest in the work amongst all sections of the staff. It was a sad disappointment to find an absence of the old spirit and atmosphere of twenty years before. We were puzzled as to the causes underlying this marked derogation from a high standard of what we might properly denominate perfection. Its absence is regrettable, and it seemed to us essential to try to discover the underlying causes which have led to this diminished efficiency.

### Some Pertinent Questions.

We refrain from stating the conclusions which our inquiries led us to arrive at, but we think it a public duty to raise certain questions in regard to this matter. Has the *personnel* or character of the committee of management materially changed during the period in question? Has there been a gradual elimination of the former principles which led some of the most distinguished residents within the county to take an active part in the management? Has there been substituted for this element a large and increasing number of less responsible persons, who have narrower views, and are inclined to run the institution more on a sectarian or narrow basis? Is there an influence in the hospital which has been gradually acquired over all the departments by someone who has not a large and generous spirit, and who may, from many years' association, have got the affairs of this institution too much into his own hands?

### The Urgent Need for Independent Investigation.

One or more of these causes might account completely for the absence of spirit, interest, and life in the place, which was so very apparent on the day of our visit. Anyway, whatever the cause may be, the time has certainly come, now that the new buildings have been completed, and this expensive work has been successfully done, that a special investigation and independent report by a hospital expert of wide experience should be ordered and presented to the governors. He could go into the whole question of the present character of the administrative arrangements with a view to the exclusion from any control over or interference with the actual management of the proper work of the hospital by any person who is not technically qualified to exercise, much less to usurp, such a position of control. We are convinced something is radically wrong at this institution. We would strongly urge the committee to keep the office, its work, and all connected with it, wholly separate and distinct from the internal management of this hospital. Let no time be lost in placing the internal administration in the hands of a resident medical officer of experience and proved ability, and the whole of the nursing and domestic affairs directly under the control of a lady superintendent of the highest standing and character, who should have the assistance of a housekeeper.

### The Buildings and Grounds.

The older portions of the buildings are attractive architecturally. The grounds are ample; they contain plenty of trees, and might be made attractive, and indeed beautiful, at very small expense. If once a little money were voted and the services of a capable landscape gardener were secured, the whole appearance of the Cumberland Infirmary, inside and out, might be changed from its present neglected and saddening aspect to one of brightness and cheer. The outlook from the ward windows is desolation, and not one foot of the ample grounds appeared to be cultivated, nor did they contain one single flower. We mentioned this point, and were told that desolation was encouraged "because a garden would cost money." It so happened that we had inspected another hospital of about the same size, with nothing like the same garden facilities, where every inch of garden space was cultivated, with the result that the institution was kept supplied during the greater part of the year with vegetables and fruit, whilst the flower beds and the general appearance of the grounds from the wards were most attractive. We commend these facts to the consideration of the committee and governors of the Cumberland Infirmary.

