# The Cumberland Infirmary, Carlisle

**Published:** 1914-10-03

**Authors:** 


### ERRATUM.

### THE CUMBERLAND INFIRMARY, CARLISLE.

In Sir Henry Burdett's Report on this Infirmary, published in The Hospital of September 19, page 677, second column, the name of Mr. Herbert W. Page was erroneously substituted for that of Dr. Henry Barnes, the consultant physician, whose life-long services to this Institution are as generally recognised as they are undoubtedly supreme. Sir Henry Burdett desires to express his regret for this unaccountable error to both Mr. Page and Dr. Barnes.

